# A Novel Tool for Distinguishing Type A Acute Aortic Syndrome from Heart Failure and Acute Coronary Syndrome

**DOI:** 10.3390/diagnostics13223472

**Published:** 2023-11-18

**Authors:** Min Joon Seo, Jae Hoon Lee, Yang-Weon Kim

**Affiliations:** 1 Department of Emergency Medicine, Dong-A University College of Medicine, Busan 49201, Republic of Korea; mj92395336@dau.ac.kr; 2 Department of Emergency Medicine, Inje University Busan Paik Hospital, Busan 47392, Republic of Korea; ywked@inje.ac.kr

**Keywords:** acute aortic syndrome, aortic dissection, acute coronary syndrome, heart failure, diagnosis, differential

## Abstract

Type A acute aortic syndrome (urgent AAS, UAAS) has a low incidence and high mortality rate; however, it is often missed or diagnosed late. Our aim was to create a new tool for distinguishing UAAS by using multiple modalities to select patients for CT aortography. This study included 75 patients with UAAS, 77 with acute coronary syndrome (ACS), and 81 with heart failure (HF) who received urgent treatment after propensity matching. Specific symptoms, past medical history, mediastinal width, region of interest (ROI) ratio in the lung base/apex, D-dimers, and troponin I were investigated to differentiate UAAS from ACS and HF. The most significant variables were selected to create a new scoring system. The UAAS score exhibited a performance AUC of 0.982. A simple UAAS score >1, excluding ROI ratios in lung base/apex, showed an AUC of 0.977, a sensitivity of 96%, and specificity of 92.41%. The results were validated using an external data set of 292 patients (simple UAAS score > 1: AUC of 0.966, sensitivity 93.33%, and specificity 95.36%). The simple UAAS score may be a valuable tool for suspecting UAAS and may reduce the likelihood of misdiagnosis or performing unnecessary CT aortography.

## 1. Introduction

The incidence of acute aortic syndrome (AAS) is very low, at 3–16 cases per 100,000 patient-years, whereas that of myocardial infarction is 200 cases per 100,000 patient-years [1]. However, the short-term mortality rates for type A (47%) and type B (13%) AAS are higher than those for myocardial infarction (8%) [1]. Nevertheless, diagnosing AAS remains a challenge due to its non-specific symptoms and the fact that it overlaps with other acute cardiovascular conditions.

There are instances where physicians may misidentify AAS as acute coronary syndrome (ACS). In particular, cases in which type A AAS (urgent AAS, UAAS) is missed can lead to fatal outcomes. The incidence of missed cases has been reported as 14–78% [2]. The main reasons for these misses are patients who present with mild symptoms or complain of anterior chest pain [3], or cases where a pulse deficit and widened mediastinum are undetected [4]. It is worth noting that mediastinal widening can also occur due to a widened vascular pedicle in patients with heart failure (HF) [5]. Additionally, more than 25% of AAS cases can be accompanied by HF [6], leading to delays in the diagnosis and treatment of AAS [7]. Therefore, physicians should recognize the need to distinguish UAAS, especially type A aortic dissection, from ACS or HF.

While computed tomography aortography (CTA) is the gold standard examination for differentiating AAS from ACS or HF, not all patients with chest pain can undergo CTA, as the prevalence of aortic dissection is only 2.5% [8]. Recently, integrating a low aortic dissection detection risk score (ADD-RS ≤ 1) and a low D-dimer (<500 ng/mL) was recommended as a rule-out criterion for AAS [9]. However, only 664 (19.4%) of 3421 patients with ADD-RS ≥ 2 or D-dimer ≥ 500 ng/mL were diagnosed with AAS [10], and many patients suspected of having AAS still undergo unnecessary CT scans.

This study aimed to identify findings of UAAS that can be distinguished from ACS and HF using baseline characteristics, chest X-rays, and laboratory findings, and to create a new discriminative tool that combines these findings.

## 2. Materials and Methods

This retrospective observational cohort study was conducted using medical records, laboratory findings, and chest X-ray data collected at a tertiary teaching hospital from January 2014 to August 2023. This study received approval from the institutional review board of our hospital (DAUHIRB-23-017). Data were anonymized and analyzed in accordance with the approved guidelines.

**Figure 1:**
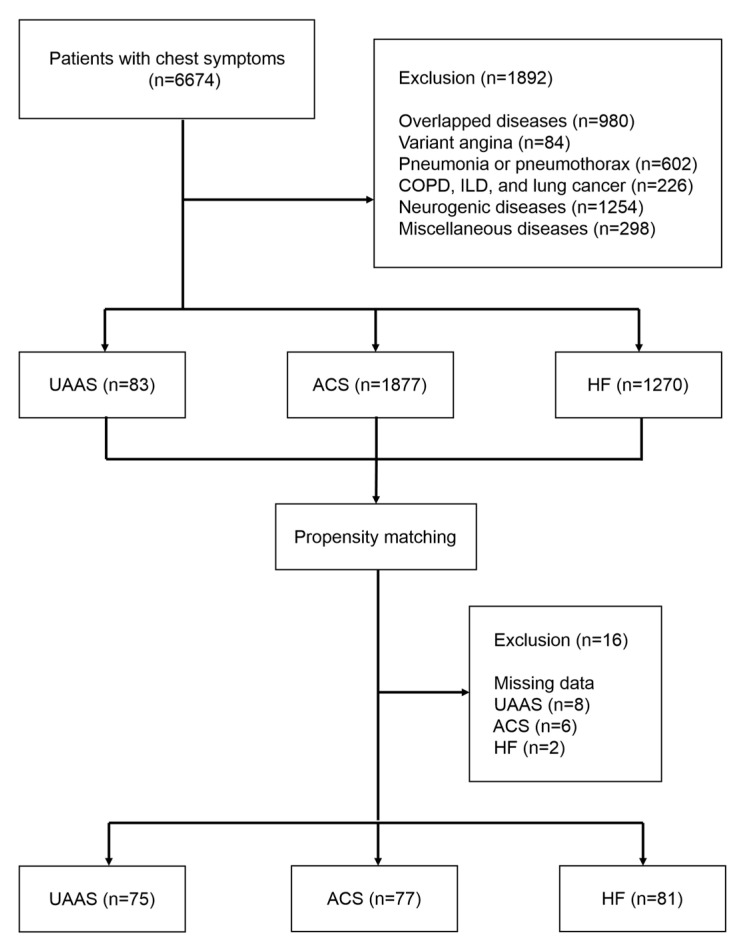
Flow sheet.

This study investigated 6674 patients who were over 18 years old, presented with acute chest symptoms, and visited the emergency department. Patients who received urgent treatment for UAAS, ACS, and HF were included. UAAS included acute aortic dissection (AAD), intramural hematoma (IMH), and penetrating aortic ulcer (PAU) that involved the ascending aorta and aortic arch and required emergency aortic lesion surgery. Excluded patients included the following categories: cases where diseases such as AAS, ACS, and HF overlapped (980 patients); ACS cases with variant angina (84 patients); cases with pneumonia or pneumothorax (602 patients); cases with diseases that could affect chest X-ray results, including chronic obstructive pulmonary disease, interstitial lung disease, and lung cancer (226 patients); neurogenic diseases (1254 patients); and miscellaneous cardiac cases with arrhythmia, cardiac tamponade, perimyocarditis, and cardiac arrest (298 patients). Consequently, patients who underwent emergency surgery for UAAS (83 patients), coronary intervention for ACS (1877 patients), and acute management for HF (1270 patients) were identified (Figure 1).

Patients who had posteroanterior (PA) chest radiographs instead of anteroposterior (AP) chest radiographs, had numerous artifacts in an AP chest radiograph, or lacked laboratory findings (UAAS, 8 patients; ACS, 6 patients; HF, 2 patients) were excluded after 1:1 propensity matching with the 83 UAAS patients based on the national early warning score (NEWS). Ultimately, the study included 75 patients with UAAS, 77 patients with ACS, and 81 patients with HF. Furthermore, an external data set was investigated in another hospital to validate the results, including 54 patients with UAAS, 119 with ACS, and 119 with HF after propensity matching and exclusion.

The following medical findings were investigated: demographics and medical history, characteristics of chest pain, vital signs, baseline laboratory findings, and NEWS. Specific characteristics of chest pain for AAS included posterior chest or back pain, acute severe pain, abrupt tearing or ripping pain, and migrating pain [11]. Previous aortic history included information regarding aortic aneurysm, coarctation of the aorta, AD, IMH, PAU, and previous aortic disease surgery.

D-dimer levels were measured using an immunoturbidimetric assay with the Sysmex CS-5100 System (Siemens Healthcare Diagnostics, Erlangen, Germany), and the reference value was <0.5 mg/L. We planned to analyze the D-dimer test results based on three different cutoff values: those determined using the Youden index, 500 ng/mL fibrinogen equivalent units, and an age-adjusted cutoff (DDadj). Troponin I levels were measured using the ARCHITECT i2000SR (Abbott Diagnostics, Lake Forest, CA, USA), and the reference value was <0.05 ng/mL. To match the severity of patient groups, NEWS was used in the propensity matching process.

A GE Optima XR220amx (GE Healthcare, Waukesha, WI, USA), a 15 kW portable indirect conversion digital radiography system with a cesium iodide detector, was used to acquire all chest radiographs for this study. The radiographs were taken in the supine AP position due to the emergency setting. All chest radiograph data were measured by a specialist.

Mediastinal width was defined as the maximal distance from the right lateral border to the left lateral border of the superior mediastinum at the level of the aortic knob. Thoracic width was defined as the maximal horizontal thoracic diameter (inner edge of ribs/edge of pleura) at the lowest level of the costophrenic angle. The region of interest (ROI) of the lung apex was measured at the darkest area of the 3rd–4th and 4th–5th intercostal spaces, and the ROI of the lung base was measured at the darkest area of the 8th–9th and 9th–10th intercostal spaces. The mediastinal/thoracic width ratio and the base/apex ROI ratio were calculated.

Continuous variables were presented as the mean value with standard deviation and analyzed using the Mann–Whitney U test. Fisher’s exact test was used to compare categorical variables. Receiver operating characteristic analysis was performed to determine the sensitivity, specificity, positive/negative likelihood ratios, and the area under the receiver operating characteristics curve (AUC) value. The performance of the clinical findings and chest radiograph parameters was compared using AUCs with optimal cutoff values. The most significant variables were selected to create a new score, and scores were assigned based on the degree of risk using cubic spline models and Youden index using ROC curves. A *p* value of <0.05 was considered statistically significant.

## 3. Results

### 3.1. Baseline Characteristics

Compared to patients with ACS or HF, patients with UAAS were younger, included a higher proportion of females, had lower mean arterial pressure, a higher incidence of back or tearing pain, a previous aortic history, lower levels of troponin I, higher levels of D-dimer, and a wider mediastinal width (Table 1). The discriminative power of back, tearing, migrating pain, or a previous aortic history had a low sensitivity (25.33%) and high specificity (98.73%) with an AUC of 0.664. Adding severe pain and prior aortic valve diseases to these pain characteristics and past history improved the diagnostic performance (AUC 0.71). ADD-RS showed significant differences in the UAAS group (*p* < 0.001). ADD-RS included pain features such as abrupt pain, severe pain, and tearing pain, and past history such as Marfan syndrome, family history of aortic disease, aortic valve disease, recent aortic manipulation, and thoracic aortic aneurysm. The performance of ADD-RS had an AUC of 0.807. The specificities for one set (including back, tearing, and migrating pain), another set (including severe and sharp pain), and prior aortic valve disease were 99.37, 91.77, and 96.84, respectively. D-dimer/troponin I and mediastinal widening were the most powerful predictors of UAAS (AUC 0.899 and AUC 0.91) (Table 2).

### 3.2. Biomarkers for UAAS Discrimination

**Figure 2:**
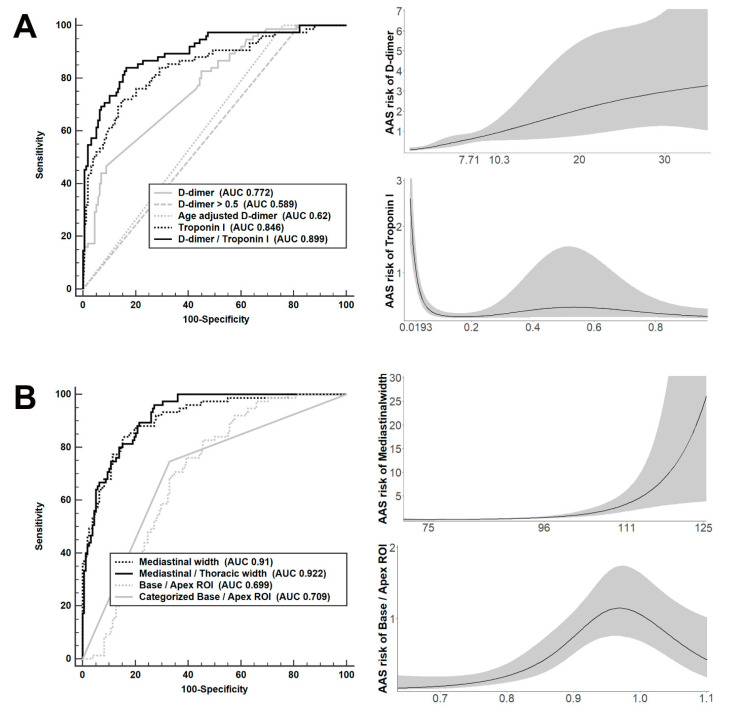
Diagnostic performance and risk assessment for distinguishing urgent acute aortic syndrome (UAAS) using various modalities. (**A**) D-dimer and troponin I. (**B**) Parameters in chest radiographs.

In laboratory findings, age-adjusted D-dimer was superior to abnormal D-dimer in distinguishing between UAAS and non-UAAS; however, the original value of D-dimer showed the best performance. We performed subgroup analysis regarding the presence of ST segment elevation and ADD-RS. The discriminative performance of D-dimer in patients with an ST elevation group (AUC 0.835) was higher than that in patients with a non-ST elevation group (AUC 0.766), and the discriminative performance of D-dimer in patients with ADD-RS ≥ 2 as a high-probability group (AUC 0.727) was lower than that in patients with ADD-RS ≤ 1 as a low-probability group (AUC 0.782). D-dimer was more increased and troponin I was more decreased in the UAAS group than in the other groups (Table 1). The threshold values of D-dimer that increased the probabilities of UAAS were 7.71 and 10.3 μg/mL, and that of troponin I was 0.0193 ng/mL (Figure 2A). Both troponin I and D-dimer were good discriminators, and the D-dimer/troponin I ratio exhibited the strongest performance in screening for UAAS.

### 3.3. Imaging for UAAS Discrimination

**Figure 3:**
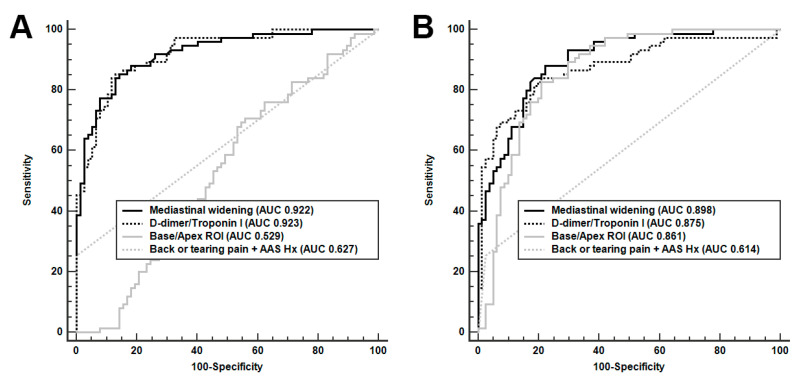
Discrimination of urgent acute aortic syndrome (UAAS) from acute coronary syndrome (ACS) and from heart failure (HF). (**A**) Discrimination of UAAS from ACS. (**B**) Discrimination of UAAS from HF.

In chest radiography findings, the mediastinal/thoracic width ratio improved the performance of distinguishing between UAAS and non-UAAS when compared to mediastinal width alone (Table 2). The threshold values of mediastinal width that increased the probabilities of UAAS were 95.8 and 110.9 mm, and those of ROI ratios in the lung base/apex were 0.88 and 1.07. The possibility of UAAS based on base/apex ROI ratios was increased at a cutoff value greater than 0.88 and decreased at values greater than 1.07 (Figure 2B). ROI ratios in the lung base/apex were an important predictor for discrimination between UAAS and HF, with an AUC of 0.861; however, this was not the case between UAAS and ACS, with an AUC of 0.529 (Figure 3). We created a new UAAS score that comprised the D-dimer/troponin I ratio (>168, 1 score; >822, 2 score), mediastinal width (>96 mm, 1 score; >111 mm, 2 score), base/apex ROI ratio (0.9 < ROI ratio < 1.1, 0.5 score), and specific symptom and history of AAS (presence of back or tearing or migrating pain or previous aortic history, 1.5 score). Mediastinal/thoracic width exhibited higher performance for the discrimination of UAAS than mediastinal width in univariable conditions; however, it showed a lower performance in multivariable conditions.

### 3.4. UAAS Score

**Figure 4:**
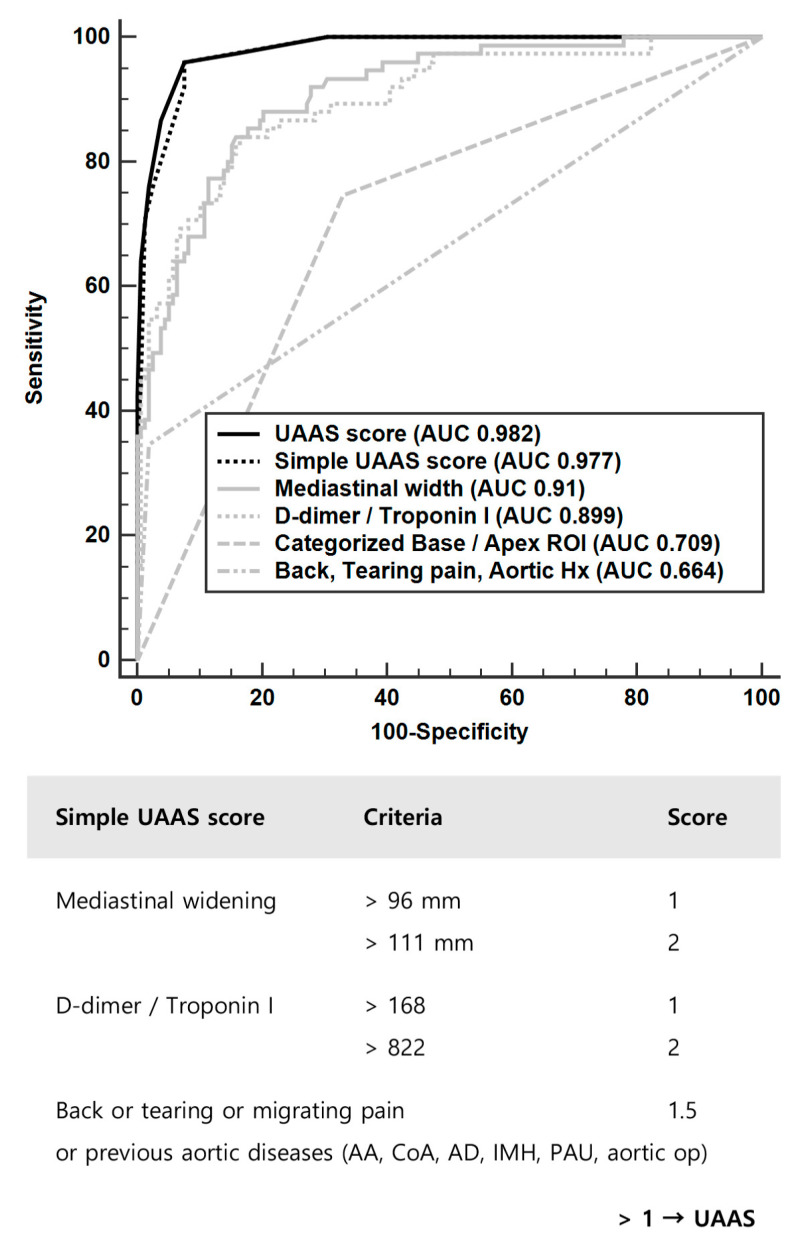
Simple urgent acute aortic syndrome (UAAS) score and comparison of staple parameters.

**Figure 5:**
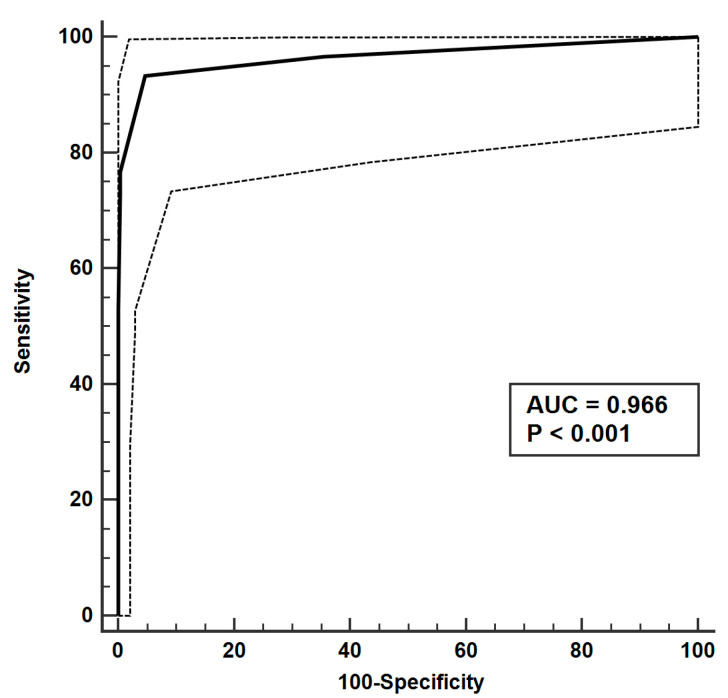
Diagnostic performance validation of simple urgent acute aortic syndrome (UAAS) score using external data set.

The performance of the UAAS score, which includes mediastinal widening, D-dimer/troponin I ratio, base/apex ROI ratio, and clinical factors such as back or tearing or migrating pain or previous aortic history, yielded an AUC of 0.982. When combining pain characteristics and past history from ADD-RS with mediastinal widening and D-dimer/troponin I ratio from the UAAS score, the AUC was slightly lower, at 0.975. The UAAS score successfully screened seventy-three out of seventy-five patients with UAAS, missing only two patients. A simple UAAS score that excludes the base/apex ROI ratio can be more available in clinical scenarios, especially when it is challenging to use the base/apex ROI ratio in patients with parenchymal lung diseases. The performance of this simple UAAS score, when greater than 1, demonstrated an AUC of 0.977, a sensitivity of 96%, specificity of 92.41%, positive predictive value of 85.7%, and negative predictive value of 98%; the components of the score are illustrated in Figure 4. For a simple UAAS score of >0, the sensitivity, specificity, positive predictive value, and negative predictive value were 100%, 69.62%, 3.29%, 0%, 61%, and 100%, respectively. Incorporating pain characteristics and past history from ADD-RS with mediastinal widening and D-dimer/troponin I ratio from the simple UAAS score resulted in a slightly reduced AUC of 0.969. The efficacy of a simple UAAS score > 1 was further validated using an external data set from another hospital, achieving an AUC of 0.966 (Figure 5). In this external validation, the sensitivity and specificity were 100% and 64.56% for a simple UAAS score of >0, respectively. If a patient presents to the emergency department with chest discomfort, without back pain, tearing sensations, migratory pain, or any past history involving the aorta, has a D-dimer level of 7.71 μg/mL, troponin I level of 0.0063 ng/mL, and mediastinal widening of 91.15 mm, physicians should suspect UAAS and consider performing a chest CT. This is based on a simple UAAS score of 2, calculated from a D-dimer/troponin I ratio of 1223.8.

## 4. Discussion

Both mediastinal widening and the D-dimer/troponin I ratio showed the best performance for discriminating UAAS. Back pain, tearing pain, and migrating pain, along with a previous history of aortic issues, were specific but not sensitive. The base/apex ROI ratio was helpful in distinguishing between AAS and HF. The simple UAAS score demonstrated high performance in UAAS screening. A simple UAAS score of >1 may be used for reasonable suspicion of UAAS and that of >0 may be used to perform CTA, preventing a missed diagnosis.

Patients with AAD have specific symptoms: back pain 28–64%, tearing pain 21.7–62%, and migrating pain 15.6–44% [1,2,12,13]. However, the symptoms are not often discovered (9.3% in our data). This may be attributed to the populational differences or incomplete medical records that were examined through a retrospective review. The simple UAAS score might have greater performance if these specific symptoms are assessed more accurately. Prior aortic valve disease, recent aortic manipulation, and prior thoracic aortic aneurysm were present in 11.9%, 14.7%, and 2.8%, respectively, of patients with AAD, similar to our data (18.7%) [14].

Compared with various factors that UAAS was associated with, mediastinal widening has rarely been studied. The diagnostic value of mediastinum widening (>8 cm) on chest radiographs appeared to be marginal in low-risk patients (ADD-RS = 0) in a prior study [15] and a widened mediastinum also played a subsidiary role in distinguishing AAS in another study [12]. Quantitative measurement of a widened mediastinum was observed in only two studies that investigated mediastinal width in supine anteroposterior chest radiographs, as in our study [16,17]. The range of widened mediastinum distinguishing AAD was 86.5–87 mm, which was different from the 95.8 mm noted in our study, which may be due to the difference in control groups (a normal group or group with mild diseases without admission in previous studies vs. the ACS and HF group with severity in our study). The quantitative measurement of a widened mediastinum should be recognized as a valuable discriminator of UAAS. Another discovery from chest radiographs was the base/apex ROI ratio, a newly developed predictor for distinguishing HF. Differences in ROIs in the lung base and apex may denote abnormal lesions of the lung parenchyma, including pulmonary edema, pneumonia, lung fibrosis, and lung mass. A base/apex ROI approaching 1 is likely to be useful in suspecting UAAS in patients with chest symptoms when acute lung diseases are not being considered.

It is well known that D-dimers maintain relatively low values in patients with ACS and high values in patients with AAD or acute pulmonary embolism [18,19]. D-dimers must be a good discriminator for UAAS diagnosis; however, in most cases, D-dimer < 500 ng/mL and DDadj are clinically used only as rule-out criteria for AAS [9]. Furthermore, a D-dimer level > 500 ng/mL has been demonstrated to be highly sensitive for acute dissection (~97%) but relatively non-specific (56%): 18% of patients with AAD had D-dimer levels <400 ng/mL [1]. Using D-dimers as rule-in criteria has limitations, but D-dimers as rule-in criteria have been studied with good performance [18].

It should be noted that disease probability and disease severity may affect diagnostic accuracy in distinguishing AAS using D-dimers. AAS patients with ADD-RS ≤ 1 tended to show lower values of D-dimers and exhibit a lower predictive power while those with ADD-RS ≥ 2 tended to show high value of D-dimers [20]. On the contrary, AAS patients with ADD-RS ≤ 1 as a low-probability group showed rather higher accuracy for AAS diagnosis in another study [19]. Furthermore, a study reported that D-dimer ≥ 5 μg/mL may differentiate acute myocardial infarction from AAD and acute pulmonary embolism, with an AUC of 0.9 [18]; other studies including only patients with ST segment elevation demonstrated that D-dimer ≥ 0.75 μg/mL and 2.155 μg/mL can distinguish AAD from acute myocardial infarction with high performance (AUCs 0.95 and 0.998) [21,22]. In our study, AAS patients with ADD-RS ≤ 1 showed a higher performance than those with ADD-RS ≥ 2, and patients with non-ST segment elevation exhibited a lower performance than those with ST segment elevation. Importantly, using the D-dimer/troponin I ratio, rather than D-dimers alone, is essential for distinguishing UAAS from ACS and HF. D-dimers should be considered in conjunction with troponin I because the level of troponin I in ACS and HF is relatively higher than that in AAS; however, this could vary based on the severity of ACS and HF. Compared with D-dimers alone, the D-dimer/troponin I ratio was more effective in distinguishing between AAS and ACS [23]. Similarly, using both D-dimers and troponin I enhanced diagnostic performance in our study.

ADD-RS is a traditional predictive tool for suspecting AAS. The performance of ADD-RS exhibited an AUC of 0.806, and when ADD-RS > 1 was combined with D-dimer > 2 μg/mL, the performance improved (AUC 0.929) [24]. In our study, the performance of ADD-RS resulted in an AUC of 0.807. Severe, sharp pain and prior aortic valve disease, which are incorporated into ADD-RS, were not included in the UAAS score. Patients with ACS and HF might frequently experience severe pain or have prior aortic dysfunction. The UAAS score, which uses back, tearing, migrating pain with higher specificity, is likely to enhance diagnostic performance compared to using severe and sharp pain or prior aortic disease with lower specificity.

Assessing UAAS through multiple modalities may be needed in various clinical scenarios. For practical utility, our study included moderate to severe diseases such as ACS and HF rather than normal subjects or mild patients as the control group. The simple UAAS score combined with multiple modalities demonstrated great performance although the ACS and HF groups in our study had a similar severity to the UAAS group. A previous history of specific symptoms, mediastinal widening, or the D-dimer/troponin I ratio may each serve as effective modalities for suspecting AAS; however, combining these three components can reduce the likelihood of missing UAAS patients who are at high risk of mortality. The UAAS score can assist physicians in assessing UAAS and deciding whether to perform a CTA.

This study has several limitations. First, this retrospective study was conducted at a single center with a small sample. However, this was because the cases of UAAS were not frequent and the population was reduced via propensity matching. Prospective and large-scale, multi-center study validation is required. Second, patients who underwent chest PA radiographs were not evaluated with the UAAS score because mediastinal width was mostly measured in chest AP radiographs. Third, disease severity may affect diagnostic accuracy in distinguishing UAAS using D-dimers and troponin I. According to the characteristics and comorbidities of control group members, the diagnostic accuracy of the D-dimer/troponin I ratio might vary. Fourth, the cutoff value of D-dimers for AAS discrimination may change based on symptom onset time [25]. Fifth, it may be challenging to apply the base/apex ROI ratio in patients with chronic lung parenchymal diseases. Sixth, this study was not conducted in a general population with various symptoms suspected of AAS. However, the majority of AAS patients have chest symptoms and distinguishing UAAS in this cohort is clinically significant. Finally, whether UAAS patients with a simple UAAS score > 0 were missed should be validated through a large, multi-center population study, despite our external validation.

## 5. Conclusions

Previous aortic history, including aortic aneurysm, coarctation of the aorta, AAD, IMH, PAU, and aortic surgery, along with specific symptoms such as back pain, tearing pain, or migrating pain, demonstrated a high specificity in diagnosing UAAS. Mediastinal widening and the D-dimer/troponin I ratio were strong indicators for UAAS diagnosis. The simple UAAS score, composed of these factors, may be an excellent tool for suspecting UAAS or for performing CT aortography without missing such patients.

## Figures and Tables

**Table 1:** Baseline characteristics.

	Urgent AAS	ACS	HF	*p* *
Age, years	65.3 ± 15.09	64.7 ± 12.05	75.7 ± 11.61	<0.001
Male, n (%)	37 (49.3)	59 (79.6)	44 (54.3)	<0.001
BMI, kg/m^2^	23.6 ± 3.74	23.4 ± 2.91	23.8 ± 4.15	0.993
Heart rate, beats/min	95.2 ± 98.54	79.3 ± 15.59	80.8 ± 17.29	0.148
MAP, mmHg	88.9 ± 19.6	97.2 ± 36.19	97.8 ± 15.17	0.01
Back or tearing pain, n (%)	7 (9.3)	0 (0)	1 (1.2)	0.003
ST segment elevation, n (%)	9 (12)	22 (28.6)	13 (16)	0.024
Previous MI, HF, n (%)	18 (24)	13 (16.9)	47 (58)	<0.001
Previous stroke, n (%)	9 (12)	5 (6.5)	17 (21)	0.025
Previous aortic diseases, n (%)	14 (18.7)	0 (0)	1 (1.2)	<0.001
eGFR, ml/min/1.73 m^2^	72.3 ± 25.01	79.4 ± 29.23	50.7 ± 29.82	<0.001
PT, %	78.2 ± 17.49	91 ± 18.08	73.3 ± 20.29	<0.001
INR	1.21 ± 0.39	1.08 ± 0.18	1.25 ± 0.28	0.001
CRP, mg/dL	2.9 ± 4.46	1.39 ± 3.3	2.08 ± 4.11	0.004
CK-MB, U/L	19.5 ± 17.76	50.4 ± 72.52	17.1 ± 10.84	<0.001
Troponin I, ng/mL	0.148 ± 0.826	8.438 ± 21.039	0.204 ± 0.6502	<0.001
D-dimer, μg/mL	12.9 ± 11.03	5.5 ± 6.7	5.1 ± 5.8	<0.001
Mediastinal width, mm	110.7 ± 15.05	85.7 ± 10.65	86.8 ± 11.53	<0.001
Thoracic width, mm	280 ± 23.84	286.9 ± 25.09	284.5 ± 27.1	0.863
ROI in lung apex, HU	2728.3 ± 2860.07	3163.8 ± 3825.66	1976.7 ± 2577.59	<0.001
ROI in lung base, HU	2503.6 ± 2407.93	3002.5 ± 3751.05	2548 ± 2767.89	<0.001
ADD-RS	1.12 ± 0.68	0.43 ± 0.59	0.17 ± 0.38	<0.001
NEWS	3.91 ± 3.08	3.93 ± 3.51	3.88 ± 3.43	0.62

* Kruskal–Wallis test.

**Table 2:** Significant discriminators for urgent AAS screening.

	Sensitivity	Specificity	+LR	−LR	+PV	−PV	AUC
Troponin I	72	84.81	4.74	0.33	69.2	86.5	0.846
D-dimer	46.67	91.14	5.27	0.59	71.4	78.3	0.772
D-dimer/troponin I	84	83.54	5.1	0.19	70.8	91.7	0.899
CK-MB	34.67	85.44	2.38	0.76	53.1	73.4	0.603
Back/tearing/migrating pain or previous AAS	34.67	98.1	18.26	0.67	89.7	76	0.664
Mediastinal width	84	84.18	5.31	0.19	71.6	91.7	0.91
Mediastinal/thoracic width	96	72.78	3.53	0.055	62.6	97.5	0.922
Base/apex ROI	82.67	54.43	1.81	0.32	46.3	86.9	0.699

+LR, positive likelihood ratio; −LR, negative likelihood ratio; +PV, positive predictive value; −PV, negative predictive value.

## Data Availability

Data available on request due to restrictions eg privacy or ethical The data presented in this study are available on request from the corresponding author.
